# A standardized scoring method for measuring white cast of mineral sunscreens and improving user compliance across diverse skin tones

**DOI:** 10.1371/journal.pone.0319891

**Published:** 2025-08-26

**Authors:** Alexandra M. Maldonado López, Emily A. Gallagher, Aiden Curry, Kylie Q. Sahloff, Ivan Domicio da Silva Souza

**Affiliations:** Good Molecules, LLC, Philadelphia, Pennsylvania, United States of America; Shiraz University of Medical Sciences, IRAN, ISLAMIC REPUBLIC OF

## Abstract

**Introduction:**

Using broad-spectrum sunscreen is an effective practice for preventing skin cancers caused by ultraviolet (UV) radiation. Mineral sunscreens containing zinc oxide (ZnO) and titanium dioxide (TiO_2_) as physical UV filters are suitable for individuals with sensitive skin or allergies to chemical UV filters. Consumer compliance with sunscreen application depends largely on its cosmetic elegance, especially regarding white cast. Despite this, there is no official method to quantify white cast and help design sunscreens for diverse skin tones.

**Methods:**

To address this gap, we developed white cast scoring protocols that combine 1) inter-rater reliability (ICC) of expert graders and thirteen study volunteers ranking the white cast produced by ZnO test formulations with 2) objective CIEL*a*b* measurements determined after sunscreen application both in vivo and in vitro.

**Results:**

Our findings demonstrate a significant correlation between increasing ZnO percentages and higher L* values (whiteness) resulting in a more visible white cast, as assessed by Pearson coefficient in vivo (r ≥ 0.822; p < 0.001) and Kruskal-Wallis (p < 0.0001) with Dunn’s post hoc pairwise comparisons. White cast scores for the ZnO formulations were substantially consistent across both in vivo and in vitro methodologies, with higher ZnO concentrations producing unacceptable levels of white cast.

**Conclusion:**

This study provides a white cast scoring system, based on subjective white cast perception and L* values, as a quantitative tool for evaluating and refining mineral sunscreen formulations, contributing to developing more cosmetically elegant sunscreens suitable for a wide range of skin tones.

## Introduction

Ultraviolet radiation (UVR) is the primary driver of several skin cancers [1]. Recent estimates state that one in five Americans will develop skin cancer in their lifetime [2]. Daily use of broad-spectrum sunscreen is an effective measure for preventing skin cancer [1,3]. Sunscreens are divided into two categories based on the presence of their UVR filter actives: chemical (also known as organic) filters or physical (also known as inorganic but commonly referred to as mineral) filters. Mineral sunscreens, which consist of the two U.S. Food and Drug Administration (FDA) approved physical filters, zinc oxide (ZnO) and titanium dioxide (TiO_2_), are safe and effective options for sun protection. In fact, ZnO and TiO_2_ have been used in sunscreens since at least the 1940s [4]. Physical filters scatter, reflect, and absorb UVR [5]. Some state that TiO_2_ effectively absorbs primarily UVB, while ZnO mainly absorbs UVA radiation, whereas combining both provides broad UVA/UVB protection [6]. Notably, at higher concentrations (> 7%), ZnO alone can provide broad-spectrum protection, as well as protection against long-wavelength UVA [7]. ZnO is a gentle option for sunscreens, making it suitable for individuals with sensitive skin or allergies to UV chemical filters.

One of the most desired properties consumers seek in sunscreen is its cosmetic elegance for an aesthetically pleasing look (the way sunscreen has easy spreadability and is invisible when applied to the skin). However, consumers tend to stray away from mineral sunscreens due to their lack of cosmetic elegance since ZnO and TiO_2_ leave a white residue on the skin, called white cast [8]. White cast caused by mineral sunscreens often deters consumers from complying with sunscreen application directions, especially those with darker complexions [9,10]. ZnO and TiO_2_ particles used in sunscreens form aggregates that can agglomerate between 0.1–10.0 μm in size [11] through their innate chemical and physical properties. The larger the agglomerate, the more direct impact on UVR absorption and the resulting sun protection factor (SPF) value [12]. Also, the bigger the agglomerate, the more obvious the white cast is [13] since more particles reflect and scatter wavelengths in the visible light range, which the retina perceives as white [5]. Thus, since ZnO and TiO_2_ particles form aggregates and agglomerates in sunscreen formulations, it is known that increasing concentrations of ZnO and TiO_2_ particles in sunscreen formulations can lead to increased white cast [8,14].

Even though there is no official method to measure white cast objectively, researchers have leveraged transparency [15] and skin color measurements [16,17] to understand white cast. This skin color assessment in various skin types can be achieved with noninvasive devices through colorimetry by analyzing the intensity of the reflected wavelength to deduce the color it is “seeing”. Colorimeters are an objective color quantification tool that represents human color vision developed under the standardization of the Commission Internationale de l’Eclairage (CIE), an international authority on light and color [18]. Their color quantification can be represented under many color systems. The 1976 CIEL*a*b* color system is widely used for more in-depth research. It operates under the premise of opponent-process theory with a three-dimensional color space. The L* axis is a grayscale with 0 (black) to 100 (white) values, which correlates with an individual’s level of pigmentation. The a* is the red-green axis that correlates with erythema because a positive a* describes red, while a negative a* describes green values. The b* correlates with pigmentation and tanning. It is the yellow-blue axis, in which a positive b* is yellow and a negative b* value is blue [19,20].

Relying on colorimetry to objectively measure white cast can be challenging to reproduce because of the variability in the size distribution, particle shape, and density of the metal oxide particles in sunscreens when spread on the skin, as well as the fact that white cast is perceived subjectively differently across various skin tones. Because of the lack of practical and validated methods for measuring white cast caused by mineral sunscreens in different skin tones, we have created a procedure for determining and recording the white cast of mineral sunscreens containing zinc oxide via CIEL*a*b* in vivo and in vitro, considering the volunteer’s perception of white cast. These methods are intended to optimize the development of mineral sunscreens by allowing the selection of formulations with a reduced white cast at the early stages of the product development cycle.

## Results

### Model fit and method validation

In vivo and in vitro experiments are essential when formulating mineral sunscreens. Clinical studies evaluate the white cast produced by mineral sunscreens across different skin tones before the product reaches the market. Meanwhile, in vitro testing offers a cost-effective method for screening and selecting promising sunscreen formulations before clinical trials. Thus, we wanted to establish protocols to reliably predict in vivo white cast from in vitro measurements. The white cast measurements in our protocols focus on the L* value of the CIEL*a*b* color system since its linear values span from black to white. For the in vivo protocol, we compared L* measurements of test formulations containing a range of ZnO (0–30%) on volunteers of different Individual Typology Angle (ITA°) subtypes (S1 Fig). When relevant, we further divided the volunteers’ ITA° subtypes into three skin pigmentation categories: **light skin pigmentation** (n = 75 L* measurements, comprising Very Light and Light ITA° subtypes), **medium skin pigmentation** (n = 45 L* measurements, comprising Intermediate and Tan ITA° subtypes), and **dark skin pigmentation** (n = 75 L* measurements, the Brown ITA° subtype only). For the in vitro protocol, we employed VITRO-SKIN over a dark or light background as the substrate for the sunscreen application (n = 30 L* measurements). The benchmark (BM) of 14.7% ZnO was not included in the model fit and validation to prevent compounding effects due to being formulated differently than the 0–30% ZnO formulations, such as having different sizes or shapes of the ZnO particles.

**Fig 1:**
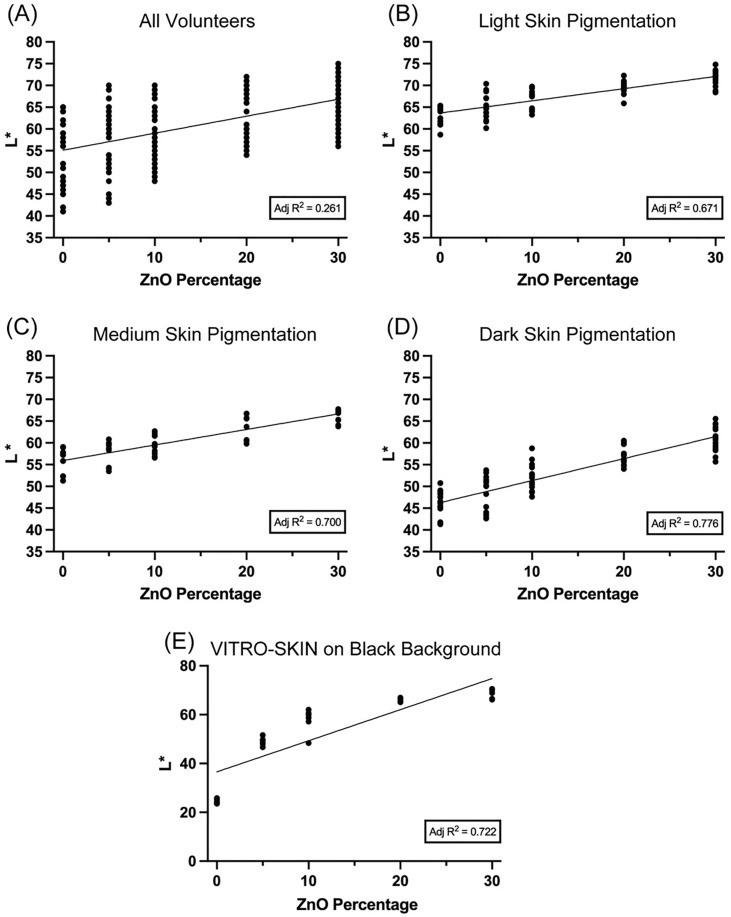
L* Value Increases with Increasing Zinc Oxide Percentage. Adjusted R^2^ was calculated to indicate the goodness of fit. (A) All Volunteers’ L* value measurements (n = 195) extend from 41 to 75. (B) Light skin pigmentation volunteers’ L* measurements (n = 75) range from 59 to 75. (C) The L* value measurements of medium skin pigmentation volunteers (n = 45) span from 51 to 68. (D) Volunteers with dark skin pigmentation have L* value measurements (n = 75) from 41 to 66. (E) VITRO-SKIN on black background L* measurements range from 24 to 71 (n = 30).

We performed linear regression analysis to investigate if the ZnO percentage explains the L* variance in the test formulations by assessing the goodness of fit in vivo and in vitro. For the analysis in vivo (Fig 1A–1D), when the measurements from all volunteers are grouped, the adjusted R^2^ of 0.261 indicates a poor fit, suggesting that the model explains only a portion of the variance in L* values based on ZnO percentage. The model’s performance improves when the data is analyzed separately by skin pigmentation categories. The fit was moderate in volunteers with light skin pigmentation, with ZnO percentage accounting for some of the variance in L* values. The model showed a good fit for medium skin pigmentation, effectively capturing most of the variance in L* and performing reliably. In the dark skin pigmentation category, the fit was particularly strong, with the model explaining the majority of the variance in L*, reflecting a robust and highly effective relationship between ZnO percentage and L*. Furthermore, the measurements on VITRO-SKIN with a black background resulted in an adjusted R^2^ of 0.722, reflecting a good fit between ZnO percentage and L* values (Fig 1E). The VITRO-SKIN on a white background had similar results (S2 Fig).

To continue our validation, a Shapiro-Wilk test was conducted to evaluate the data distribution before performing a correlation analysis between the L* values and ZnO percentages. Since our in vivo data were normally distributed, we performed Pearson correlations to determine the strength of the correlation between ZnO percentage and L* (Table 1). Interestingly, when the volunteers’ measurements were evaluated altogether, there was a moderate correlation between ZnO percentage and L* value, r = 0.520 (p < 0.001). Then, we calculated the Pearson correlation for the three skin pigmentation categories. Light, medium, and dark skin pigmentation volunteers had an r ≥ 0.822 (p < 0.001), indicating strong correlations between ZnO percentage and L* when analyzed per skin pigmentation group. An increase in correlation (r-value) was observed with higher skin pigmentation (a darker background for the white cast). For the in vitro measurements, because they were not normally distributed, we performed a Spearman rho analysis (Table 1), resulting in a strong correlation between the ZnO percentage and the L* values, ρ ≥ 0.938 (p < 0.001).

**Table 1:** Model Performance and Linearity Metrics In Vivo and In Vitro.

Parameter	All Volunteers	Light Skin Pigmentation	Medium Skin Pigmentation	Dark Skin Pigmentation	Black Acrylic	White Acrylic
Linearity Range (ZnO %)	0 - 30	0 - 30	0 - 30	0 - 30	0 - 30	0 - 30
Regression Equation	y = 0.3910x + 55.11	y = 0.28x + 63.7	y = 0.357x + 55.9	y = 0.506x + 46.3	y = 1.27x + 36.6	y = 0.0922x + 87.2
Slope ± SD	0.3910 ± 0.04625	0.2801 ± 0.02270	0.3570 ± 0.03506	0.5057 ± 0.03311	1.274 ± 0.1458	0.09224 ± 0.008862
Intercept ± SD (L*)	55.11 ± 0.7808	63.66 ± 0.3832	55.94 ± 0.5918	46.28 ± 0.5550	36.59 ± 2.461	87.22 ± 0.1496
Pearson (p < 0.001)	0.520	0.822	0.841	0.883		
Spearman rho (p < 0.001)					0.938	0.951
n	195	75	45	75	30	30

We wanted to ensure that the measurements made by the NYX Pro 2 Color Sensor colorimeter were accurate and precise. In vivo, accuracy was calculated by comparing the L* values of all ITA° subtypes present in the study (Very Light to Brown subtypes) to the mean values of L* for ITA° subtypes previously published [16]. All accuracy (Table 2) was between 88–96% of the reference values. Furthermore, precision calculations were made for all ITA° subtypes and the three subsets of Light, Medium, and Dark skin pigmentation (Tables 3 and S1). The Relative Standard Deviations (RSDs) of the L* before and after the sample application were < 10%. In vitro, accuracy was analyzed by measuring CIEL*a*b* of pure black and white standard paints on VITRO-SKIN with a black background in comparison with CIEL*a*b* measurements of theoretical pure black (L* = 0, a* = 0, b* = 0*) and theoretical pure white (L* = 100, a* = 0, b* = 0). The accuracy of measuring black on a black acrylic background was 89%, while for white, it was 93% (Table 2). We also calculated the inter- and intra-day precisions (Tables 3 and S2) of our L* measurements on VITRO-SKIN with black acrylic as the background, resulting in RSDs < 7% before and after the test formulation application. The same measurements were taken and analyzed for VITRO-SKIN on a white background, yielding similar results (Tables 2, 3 and S2). To complement these in vitro quantitative assessments, we also captured photographs of VITRO-SKIN before and after test formulation application on both black and white backgrounds to observe color changes qualitatively (S3 and S4 Fig). Notably, the white background made it more difficult to perceive changes in white cast, whereas the black background provided greater visual contrast.

**Table 2:** Accuracy of L* Measurements for the In Vivo and In Vitro Protocols.

Factor		Accuracy (%)^a^
In vivo Results	Very Light Subtype ITA°	88.11 [81.60 - 94.62]
	Light Subtype ITA°	94.62 [92.20 - 97.04]
	Intermediate Subtype ITA°	94.99 [93.05 - 96.92]
	Tan Subtype ITA°	95.34 [93.74 - 96.95]
	Brown Subtype ITA°	95.52 [91.31 - 99.72]
In vitro Results	Black Paint on Black Acrylic	89.16 [87.80 - 90.52]
	White Paint on Black Acrylic	92.53 [86.98 - 98.07]
	Black Paint on White Acrylic	88.26 [86.39 - 90.14]
	White Paint on White Acrylic	90.39 [81.97 - 98.81]

^a^Results are expressed in Mean [C.I._95%_].

**Table 3:** Precision of L* Measurements for the In Vivo and In Vitro Protocols.

Factor		% RSD^a^ Before Sample Application	% RSD^a^ After Sample Application
Inter-Day In Vivo	Very Light Subtype ITA°	0.57% ≤ RSD ≤ 1.48%	0.40% ≤ RSD ≤ 1.56%
	Light Subtype ITA°	2.41% ≤ RSD ≤ 4.31%	2.23% ≤ RSD ≤ 5.02%
	Intermediate Subtype ITA°	1.05% ≤ RSD ≤ 2.81%	1.37% ≤ RSD ≤ 5.31%
	Tan Subtype ITA°	0.73% ≤ RSD ≤ 2.80%	0.76% ≤ RSD ≤ 2.45%
	Brown Subtype ITA°	6.46% ≤ RSD ≤ 9.28%	3.51% ≤ RSD ≤ 8.55%
	Light Skin Pigmentation	2.2% ≤ RSD ≤ 3.87%	2.07% ≤ RSD ≤ 4.52%
	Medium Skin Pigmentation	4.18% ≤ RSD ≤ 5.66%	3.35% ≤ RSD ≤ 5.92%
	Dark Skin Pigmentation	6.46% ≤ RSD ≤ 9.28%	3.51% ≤ RSD ≤ 8.55%
Inter-Day In Vitro	Black Acrylic	3.20% ≤ RSD ≤ 6.41%	1.11% ≤ RSD ≤ 4.76%
	White Acrylic	0.39% ≤ RSD ≤ 0.73%	0.24% ≤ RSD ≤ 0.55%
Intra-Day In Vitro	Black Acrylic	0.71% ≤ RSD ≤ 4.30%	0.12% ≤ RSD ≤ 6.85%
	White Acrylic	0.08% ≤ RSD ≤ 1.15%	0.05% ≤ RSD ≤ 0.78%

^a^All RSDs were reportedly < 10%.

### Perceived whiteness across skin tones

**Fig 2:**
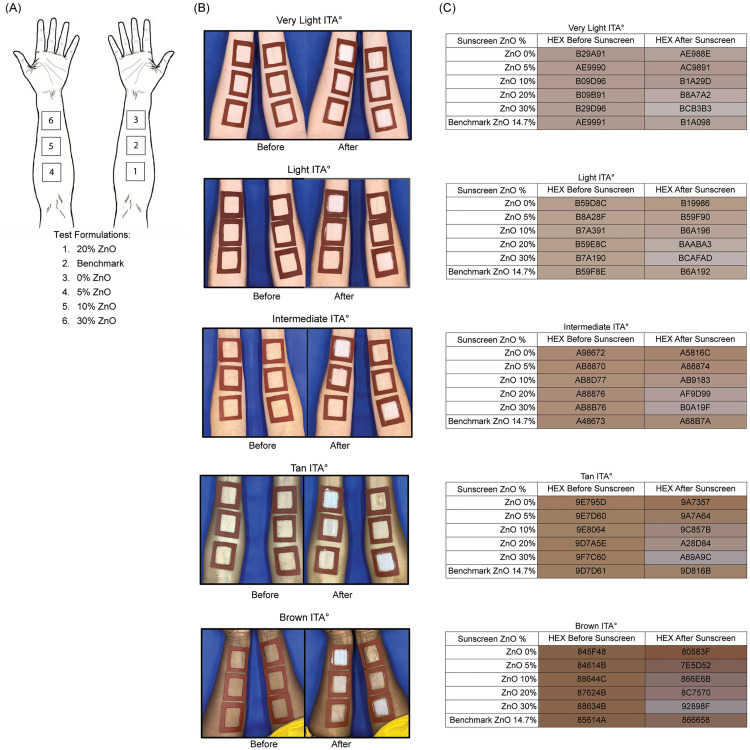
White Cast Visualization Caused by Different Zinc Oxide Percentages in a Variety of Skin Tones. (A) Legend indicating which formulation was applied to each square on the volunteers’ forearms. (B) Representative images of how white cast caused by different zinc oxide percentages are experienced by each ITA° subtype. (C) Average HEX skin color of each ITA° subtype before and after the application of each test sample.

The in vivo study was carried out with a panel of thirteen (13) female volunteers, aged 20–60 years, Fitzpatrick II-VI, with diverse ethnicities (including 31% Caucasians and 38% Black or African Americans, S3 Table). We recruited volunteers based on their Fitzpatrick Skin Type but analyzed the research based on the volunteers’ ITA° subtypes (n = 13) (S1 Fig). When relevant, we further divided the volunteers’ ITA° subtypes into three skin pigmentation categories: **light skin pigmentation** (n = 5 volunteers, comprising Very Light and Light ITA° subtypes), **medium skin pigmentation** (n = 3 volunteers, comprising Intermediate and Tan ITA° subtypes), and **dark skin pigmentation** (n = 5 volunteers, the Brown ITA° subtype only). Photographs and CIEL*a*b* measurements were taken of the volunteer’s skin before and after each test formulation application to observe how the skin color changed subjectively (Fig 2). To understand the color variation, we converted the mean CIEL*a*b* measurements into Hexadecimal color codes (HEX) for more convenient observation. In the representative pictures of each ITA° subtype (Fig 2B), we noticed that in the Very Light, Light, and Intermediate ITA° subtypes, the 30% ZnO sample exhibits a prominent white cast on their skin, while, in comparison, the 20% ZnO caused less white cast. In the Tan and Brown ITA° subtypes, the 20% and 30% ZnO samples caused a more apparent white cast, but the 10% ZnO also produced some white cast on their skin tones (Fig 2B). Observing the HEX color values (Fig 2C), a grey gradient is noticed with the increase in ZnO percentage. The grey gradient can be observed in the Very Light, Light, and Intermediate ITA° with the 20% and 30% ZnO samples, while it is more striking in the Tan and Brown ITA° subtypes, starting at the 10% ZnO (Fig 2C).

Next, we confirmed these findings with expert grading. A panel of trained white cast graders independently ranked the post-application photographs for every volunteer and VITRO-SKIN on a black background from least to most visible cast. The formulations with the lowest ZnO content were consistently rated as producing the least white cast, whereas higher ZnO concentrations were consistently judged to cause more white cast (Table 4). An exception to this was the benchmark, which may include additional technologies to disguise white cast. Interclass Correlation Coefficient (ICC) was calculated to explore the consistency of the ranking between expert graders (ICC = 0.992, CI_95%_ = 0.989–0.995; p < 0.001), confirming that expert perception closely follows the objective color shifts observed in Fig 2C.

**Table 4:** Three Experts and 13 Volunteers Show Agreement in Ranking White Cast from Least to Most with Increasing ZnO Percentage.

Test Formulation	Expert Grading Rank (Median [Q1, Q3])	Volunteer Subjective Rank (Median [Q1, Q3])
ZnO 0%	1 [1, 1]	1 [1, 1]
BM ZnO 14.7%	2 [2, 2]	2 [2, 3]
ZnO 5%	3 [3, 3]	3 [2, 3]
ZnO 10%	4 [4, 4]	4 [4, 4]
ZnO 20%	5 [5, 5]	5 [5, 5]
ZnO 30%	6 [6, 6]	6 [6, 6]
	ICC = 0.992 (CI_95%_ = 0.989–0.995; p < 0.001)	ICC = 0.991 (CI_95%_ = 0.975–0.999; p < 0.001)

### L* measurements

**Fig 3:**
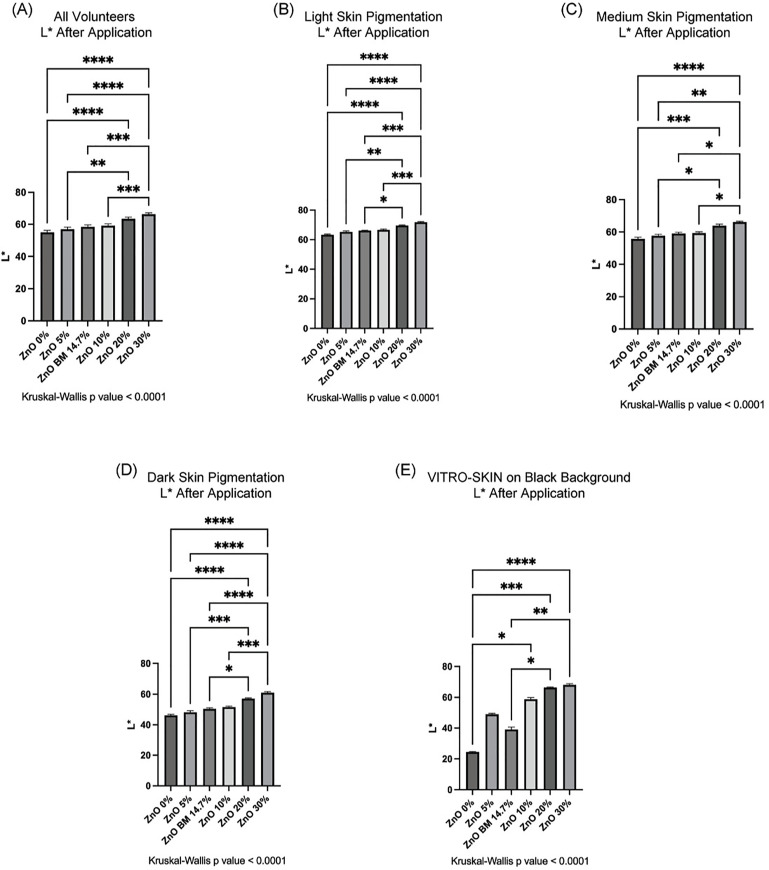
L* Value Increases with Higher Concentrations of Zinc Oxide in Formulations. In vivo: (A) 20% and 30% ZnO test formulations show statistically significant changes in L* values after application in All Volunteers in comparison to 0%, 5%, BM and 10% ZnO test formulations (** p < 0.01, *** p < 0.001 and **** p < 0.0001, n = 39). (B-D) Light skin (n = 15), medium skin (n = 9) and dark skin pigmentation (n = 15) subcategories show statistically significant changes in L* value after using 20% and 30% ZnO test formulations when compared to test formulations with lower ZnO percentages (0%, 5%, 10%, and BM) (* p < 0.05, ** p < 0.01, *** p < 0.001 and **** p < 0.0001). (E) In vitro: 20% ZnO test formulation shows a statistically significant difference after its application when compared to BM and 0% ZnO formulations (* p < 0.05 and *** p < 0.001, respectively). And the test formulation with 30% ZnO shows statistically significant changes in L* values after its application in comparison to BM and 0% ZnO formulations (** p < 0.01 and **** p < 0.0001, respectively). n = 6. Error bars are shown as mean ± SEM.

Apart from subjectively visualizing the white cast difference caused by each test formulation, we quantified the whiteness (L* value) change caused by each ZnO percentage (Fig 3). Kruskal-Wallis tests with Dunn’s post hoc pairwise comparisons were done to analyze all test formulations and determine differences in L* measurements among them. A Wilcoxon Signed Ranked test was performed to determine the objective shift in whiteness caused by ZnO percentage before and after the test formulation application (S5 Fig). All volunteers had a significant increase in L* value after applying the 30% ZnO test formulations compared to the 0%, 5%, BM and 10% ZnO formulations, indicating that the 30% ZnO formulation produces more whiteness across all skin tones (Fig 3A). Since we observed subjectively that the formulations do not produce the same white cast in different skin tones, and the baseline of L* of each subcategory spans different L* values [26], we decided to continue our analysis on the three skin pigmentation categories: light, medium and dark. When analyzed by subcategory, we noticed that the L* value associated with the 20% and 30% ZnO formulations, compared to the 0%, 5%, BM and 10% ZnO formulations was more statistically significant in light and dark skin pigmentation volunteers than on those volunteers in the medium skin pigmentation subcategory. This was especially evident when comparing the 30% ZnO formulation to the 5% and 10% ZnO formulations (p < 0.0001 and p < 0.001, respectively), (Fig 3B–D). Similar to the in vivo study, we quantified the whiteness shift caused by each ZnO percentage on VITRO-SKIN with a black acrylic sheet as the background (Fig 3E). Interestingly, the BM test formulation has a lower L* value than the 5% ZnO, which is opposite to the in vivo data across all skin tone groups. The 20% and 30% ZnO formulations caused statistically significant increases in L* compared to the BM, whereas the 5% ZnO formulation did not differ significantly from either the 20% or 30% ZnO formulations (Fig 3E).

### Impact of subjective white cast perception

A subjective questionnaire (S1 Appendix) was administered to gauge the volunteers’ opinions on white cast, resulting in 85% expressing concerns about white cast on their skin when applying sunscreen. Questions about the test formulations were also administered. The volunteers ranked the formulations on their forearms from least to most white cast. An ICC was calculated for their ranking to explore if there was consistency in how the volunteers perceived white cast on their skin. Volunteers had difficulty discerning the white cast between the 5% ZnO and BM 14.7% ZnO formulations. Still, the test formulations with the lowest ZnO content were consistently ranked as producing the least white cast. In contrast, higher ZnO concentrations were consistently judged to cause more white cast, in agreement with the expert graders (Table 4). Nonetheless, we obtained an ICC of 0.991 (p < 0.001) with a 95% confidence interval between 0.975 and 0.999, indicating a remarkably high level of ranking agreement amongst volunteers. This high level of agreement provides a foundation for interpreting the responses to the subsequent questions (Table 5). When asked about the maximum level of white cast they would accept on their face in exchange for sun protection, 23% of volunteers preferred a sunscreen with 0% ZnO, 38% were comfortable with 5% ZnO or the BM sunscreen, 31% preferred 10% ZnO and only one volunteer was willing to accept a 30% ZnO white cast. The preferences shifted when the same question was asked regarding white cast on the body. Only one volunteer preferred 0% ZnO, 31% were comfortable with 5% ZnO or the BM, 38% preferred 10% ZnO, and 23% were open to sunscreens with a 20% to 30% ZnO level of white cast.

**Table 5:** White Cast Subjective Assessment.

Factor		n	%
Are you concerned about white cast?	Strongly Agree	7	53.9
	Agree	4	30.8
	Disagree	2	15.4
	Strongly Disagree	0	0.0
What level of white cast on the face are you willing to trade for sun protection (SPF)?	0% ZnO	3	23.1
	5% ZnO	3	23.1
	10% ZnO	4	30.8
	20% ZnO	0	0.0
	30% ZnO	1	7.7
	14.7% ZnO Benchmark	2	15.4
What level of white cast on the body are you willing to trade for sun protection (SPF)?	0% ZnO	1	7.7
	5% ZnO	1	7.7
	10% ZnO	5	38.5
	20% ZnO	1	7.7
	30% ZnO	2	15.4
	14.7% ZnO Benchmark	3	23.1
n = 13			

### White cast score

**Fig 4:**
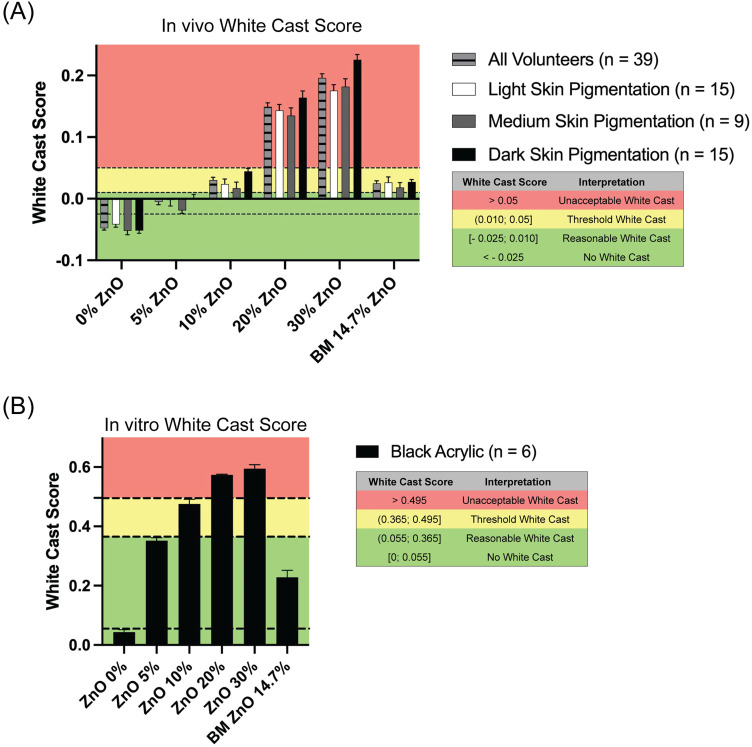
In vivo and in vitro White Cast Scores. Scores are categorized as “No White Cast”, “Reasonable White Cast”, “Threshold White Cast” and “Unacceptable White Cast” based on a combination of L* values and volunteer feedback. (A) White cast scores determined for in vivo measurements. (B) White cast scores determined for in vitro measurements. BM = benchmark. Error bars are shown as mean ± SEM.

There is currently no official method to quantify the amount of white cast caused by sunscreens. To address this gap in sunscreen formulation, we developed and proposed a white cast score specifically for mineral sunscreens. The white cast score for each test formulation was calculated and normalized according to the equation: $White\;Cast\;Score\;=\;\frac{L*f\;-\;L*i}{100\;-\;L*i}$ where L*f_* *_=  L* measurement after test formulation application, and L*i  =  L* measurement before test formulation application. The white cast scores for each test formulation and protocol are presented in a graph along with their interpretation (Fig 4)*.*

For in vivo (Fig 4A), the negative control test formulation with 0% ZnO showed no white cast on any volunteer. The 5% ZnO test formulation produced a reasonable white cast in all volunteers. The 10% ZnO and the BM (14.7% ZnO) formulations in the majority of skin tones fell into the threshold white cast category, between reasonable and unacceptable white cast levels. Finally, 20% and 30% ZnO formulations received an unacceptable white cast score for all volunteers. For in vitro (Fig 4B), the 0–30% ZnO formulations essentially replicated the in vivo scores. The BM sunscreen differed from the in vivo results by being classified as a reasonable white cast in vitro.

Using the Pearson correlations obtained during our model fit and validation tests, we verified whether the correlation coefficients for each in vivo category differed significantly from the in vitro coefficient after normalization (Table 6). Results showed no significant differences between the normalized correlation coefficients for any of the comparisons: All volunteers vs. VITRO-SKIN on black background, Light skin-pigmented volunteers vs. VITRO-SKIN on black background, Medium skin-pigmented volunteers vs. VITRO-SKIN on black background, and Dark skin-pigmented volunteers vs. VITRO-SKIN on black background.

**Table 6:** White Cast Scores for In Vivo and In Vitro Protocols Correlate Equivalently.

Comparison	Pearson Correlations (p < 0.001)	Fisher’s Z Conversion	Z difference^a^
All Volunteers vs. Black Acrylic	0.904 vs. 0.774	1.494 vs. 1.030	1.315
Light Skin Pigmentation vs. Black Acrylic	0.884 vs. 0.774	1.394 vs. 1.030	0.946
Medium Skin Pigmentation vs. Black Acrylic	0.919 vs. 0.774	1.583 vs. 1.030	1.312
Dark Skin Pigmentation vs. Black Acrylic	0.928 vs. 0.774	1.644 vs. 1.030	1.520

^a^Z differences < 1.96 are not statistically significant at the 0.05% level.

## Discussion

To address the lack of a standardized method for quantifying white cast in sunscreen formulation, we developed a standardized protocol to measure the white cast caused by mineral sunscreens both in vivo and in vitro using CIEL*a*b* measurements. In vivo, melanin content in the epidermis correlates negatively with L* values. Therefore, individuals with lighter skin pigmentation have a higher L* value and a higher ITA° color classification than individuals with darker skin tone [18,21–23]. Recognizing this, we divided the volunteers into the light, medium, and dark skin pigmentation categories due to the different baseline L* values for each ITA° subtype. This categorization allowed us to better evaluate the performance of various sunscreen formulations across diverse skin tones.

Formulations used for testing containing 20% and 30% ZnO caused significant white cast visually and quantitatively in light, medium, and dark skin pigmentation categories. Interestingly, the 10% ZnO test formulation exhibited white cast visually in photos of Tan and Brown subtypes despite the absence of statistical significance in the L* shift. This observation positioned the 10% ZnO test formulation within a critical range for white cast quantification. Additionally, the 5% ZnO and BM test formulations are particularly intriguing, as there is no statistically significant difference in the L* shift after their application, and regardless of skin tone, volunteers could not visually distinguish between the 5% ZnO and BM formulations. However, expert graders could distinguish subtle differences between these two test formulations, indicating that trained assessment may detect white cast changes not perceived by untrained observers. This highlights the complex interrelationship between measurable white cast and consumer perception. Notably, in vitro measurements using VITRO-SKIN with black acrylic background detected significant L* value changes across all ZnO concentrations, confirming its sensitivity and reliability.

To complement these objective white cast measurements obtained, we administered a subjective questionnaire to volunteers to evaluate consumer preferences and needs regarding the cosmetic elegance of sunscreens. Volunteers prefer formulations with minimal white cast on their faces when choosing sunscreen, while they are more willing to tolerate white cast on their bodies if the sunscreen provides effective sun protection. This difference in the preference for white cast on the face versus the body can be due to aesthetics and cosmetic elegance. A volunteer expressed this sentiment: “It [white cast by mineral sunscreens] does not bother me too bad on the rest of my body, but it is quite obvious on my face”.

These insights, along with the L* value measurements, informed our development of a white cast score with four categories: “no white cast”, “reasonable white cast”, “threshold white cast”, and “unacceptable white cast”. The threshold white cast category is a borderline range that falls between being reasonable (cosmetically acceptable) and unacceptable (not aesthetically pleasing). Sunscreen formulations with a white cast score in this range function like an ambiguous element that may be subjectively perceived as either reasonable or unacceptable, depending on skin tone and individual preferences. Our analysis showed that the 0–30% ZnO test formulations had consistent white cast scores in vivo and in vitro, with the higher ZnO percentages having unacceptable white casts (20–30%). However, the 5% ZnO and BM test formulations fall into different scores in vivo (reasonable vs threshold white cast, respectively), even though volunteers could not visually distinguish between them. This highlights the discrepancy between objective measurements and the consumer’s perception. Interestingly, the in vitro method proved more effective in identifying the BM test formulation as having a reasonable white cast score, aligning more closely with the expert grading ranking of test formulations and the volunteer preferences in white cast on their skin. Moreover, no significant difference between the direction of in vivo and in vitro white cast scores was observed, indicating that the white cast scores for both protocols correlate equivalently. These results emphasize the in vitro method as a reliable tool for predicting consumer perception when optimizing sunscreen formulations.

We have developed a validated protocol for measuring and recording the white cast of mineral sunscreens containing ZnO with all in vivo and in vitro L* achieving > 88% accuracy and being satisfactorily precise, with all RSDs < 10%. This white cast measurement protocol can assess white cast in vivo for a finished formulation or in vitro while formulating mineral sunscreens containing ZnO, and potentially other white pigments, such as TiO_2_. Implementing this protocol requires consistent lighting and careful calibration of the colorimeter to ensure accurate results that accommodate diverse skin types and ethnicities. Our study has provided valuable insights; however, it is essential to note its limitations. When measuring the ZnO 0% test formulation in vitro with black acrylic as background, statistically significant differences exist between the L* values when comparing the before and after measurements (p < 0.01). This suggests that the other ingredients in the sunscreen formulations, not UV filters, may also affect the L* values. Additionally, we used one ZnO ingredient among the many types available for formulating. Different kinds of ZnO particles produce different white casts due to the manufacturing process altering their shapes, sizes, and transparency. For example, the BM ZnO sunscreen scored as threshold white cast in vivo but as reasonable white cast in vitro. This discrepancy may be due to how the ingredients of the BM formulation interact with the VITRO-SKIN model. Another limitation lies in our volunteer pool and size (a total of thirteen volunteers); even though the in vivo study proved successful in measuring white cast in a wide range of skin types in the ITA° objective color classification, we could not recruit volunteers with a Dark ITA° subtype, which limits the generalizability of the findings. This limitation originates from the historical inconsistency of the Fitzpatrick Skin Type classification, which was originally developed for lighter skin tones. The Fitzpatrick scale relies on subjective evaluations of hair and eye color alongside responses to ultraviolet radiation exposure, making it less reliable for darker skin tones [24,25]. In contrast, ITA° offers an objective and quantitative approach to skin color classification, making it more suitable for dermatological and cosmetic research. For instance, while we did not classify any volunteer as a Fitzpatrick skin type I, there is a Very Light ITA° subtype volunteer. The same is observed inversely; two volunteers have a Fitzpatrick skin type of VI, but our analysis has no Dark ITA° subtype volunteers.

In summary, our protocol demonstrates a clear correlation between the increase in white cast and the ZnO percentage in sunscreen formulations across different skin tones. These findings reinforce the objectivity of ITA° and support its continued use alongside CIEL*a*b* measurements for more reliable assessments [19]. The key observation is that white cast manifests differently depending on skin tone. This challenges the common assumption that only darker skin tones struggle with white cast; white cast manifests differently across skin tones but can be equally undesirable. The cosmetic industry must account for various skin tones when formulating mineral sunscreens. While some sunscreens provide reasonable white casts that are acceptable and aesthetically pleasing for certain skin tones, they often fail to achieve cosmetic elegance for others, leading to a critical gap in inclusivity within sunscreen development. Understanding and addressing consumer’s concerns about cosmetic elegance is essential to ensure the satisfactory and consistent use of sunscreens.

## Conclusion

In this study, we have developed a standardized procedure for measuring and recording the white cast of mineral sunscreens containing ZnO in vivo and in vitro. This practical methodology, which involves objectively quantifying the white cast through colorimetry analysis and subjectively assessing it by photography and questionnaire, is designed to be easily applied in the development of mineral sunscreens. This procedure intends to optimize product development, allowing the early selection of formulations with a reduced white cast and enhanced cosmetic elegance for an improved consumer experience and, hopefully, user compliance.

## Materials and methods

### Test material

Preparations containing various percentages of zinc oxide (ZnO) were used for the studies presented in this paper. Five test formulations containing 0.00%, 5.00%, 10.00%, 20.00%, or 30.00% ZnO were made in our laboratory (S4 Table). The ZnO used in these formulations is coated with triethoxycaprylylsilane, which has a particle size > 100 nm and was obtained from BASF (Florham Park, New Jersey, USA). The FDA allows up to 25% ZnO for over-the-counter sunscreen products for human use according to the Code of Federal Regulations: 21 CFR §352.10 [26], but one of our formulations contained 30% ZnO. This was done according to the International Council for Harmonisation of Technical Requirements for Pharmaceuticals for Human Use (ICH) reportable ranges for common uses of analytical procedures, which states that for an assay of a drug substance or a finished (drug) product, the high-end of the reportable range is 120% of declared content or 120% of the upper specification limit [27]. The five test formulations were sent to contract laboratories for analytical assay of their ZnO percentage as determined by Inductively coupled plasma-optical emission spectroscopy (ICP-OES), and each formulation was confirmed accurate to the theoretical target. The test formulations were also sent for microbiological assay of total plate count, yeast and mold, coliforms, *E. coli*, *Pseudomonas* spp., *S. aureus*, *Salmonella* spp., and *C. albicans* (USP 61 and USP 62). There was no microbiological contamination present. Additionally, a primary dermal irritability 48-hour patch test study was conducted with 65 volunteers, and the test formulations did not elicit irritation. A commercially available Broad Spectrum SPF 30 with 14.7% ZnO and no other UV filter was used as the benchmark (BM) sample for comparison (S5 Table). For the in vivo and in vitro studies presented, each test formulation (a total of six) was applied at the clinically recommended dose of 2 mg/cm^2^.

### Colorimetry

The NIX Pro 2 Color Sensor (NixSensor, Hamilton, Ontario, Canada), along with the Nix Toolkit App available for iPhone, was the colorimeter used in this study for both in vitro and in vivo CIEL*a*b* measurements [28,29]. The suitability of the NIX Pro 2 Color Sensor was checked daily before any experiment by conducting triplicate color measurements on black and white acrylic sheets (200 x 300 x 2 mm). The L*, a*, and b* relative standard deviations (RSD) were expected to be ≤ 2.0%.

### In vivo protocol for white cast measurement

This protocol was a closed-label, double-blind study investigating how much white cast different ZnO formulations can cause in various skin types. Good Molecules, LLC, USA, sponsored and conducted this study between September and October 2024. Recruitment of potential volunteers was done between September 3^rd^, 2024 – October 1^st^, 2024, via email or text message by sending a flyer with a description of the study, basic requirements, and contact information to apply for the screening and subsequent participation in the study. The protocol complied with the guidelines of the Declaration of Helsinki and was approved by the Allendale Investigational Review Board (Old Lyme, CT, USA), which ensures that the study is conducted ethically and per regulatory requirements (Study Number GMCS#1–2024 approved on August 29th, 2024). All volunteers signed a written informed consent before initiating the study.

Healthy volunteers of legal age with a Fitzpatrick skin type between II and VI who were willing to comply with the procedures, methods, evaluations, and test product usage described in the written informed consent were included in the study. Volunteers were excluded if they had 1) dermatological conditions in the arms, such as Vitiligo, Rosacea, and Contact dermatitis, due to the potential interference with the study’s measurements; 2) a history of melanoma or treated skin cancer within the last five years due to the potential risk of skin damage; 3) tattoos, moles, or birthmarks covering the majority of their forearms due to the potential difficulty in measuring the white cast; 4) hairy forearms and an unwillingness to shave for the study due to the possible interference with the study’s measurements; and 5) a known allergy to any ingredient in the test formulations to ensure the safety of the volunteers and the accuracy of the study’s results.

Fourteen healthy adult volunteers started the study following the screening and recruitment process. However, one volunteer was excluded from the data analysis due to an ingredient allergy that prevented testing the benchmark sample and for not following the protocol. No other protocol deviations occurred during or after the study. The volunteers arrived at the study site with bare forearms, clean of skin care products, including sunscreen and moisturizers. Before measurements, the volunteers were given 15 minutes to acclimate to the ambient conditions. The research staff then cleaned the volunteers’ forearms with saline wipes, where they fixed six 3.0 x 3.0 cm silicone squares with hypoallergenic adhesive tape - one square per test formulation. Three baseline CIEL*a*b* measurements were taken at the top left, middle, and bottom right inside each silicone square, as per S6 Fig. The mean Individual Typology Angle (ITA°) was calculated from the CIEL*a*b* baseline measurements to categorize the volunteers’ skin color. The research staff then applied 2 mg/cm^2^ of each test formulation inside its respective silicone square (Fig 2A). After application, a 15-minute rest period was observed to let the formulations set on their skin before repeating CIEL*a*b* measurements.

During the study, the volunteers also completed an ad hoc subjective questionnaire designed to collect their opinion on white cast and the test samples (S1 Appendix). The subjective perception of white cast collected with this questionnaire was used to develop the white cast score proposed herein. A flowchart summarizing the methodology can be found in S3 Appendix.

### Subjective ranking analysis

Participants completed a four-item questionnaire to evaluate their perception of white cast, personal preferences regarding white cast, and impressions of the test formulations applied to their forearm skin (S1 Appendix). Question 4 specifically asked participants to rank the test formulations from least to most white cast by using a continuous line scale. These rankings were analyzed to assess inter-rater reliability. Intraclass correlation coefficients (ICC) and their 95% confidence intervals were calculated using SPSS Version 30.0.0 (IBM Corp., Armonk, NY, USA), employing a two-way mixed-effects model with absolute agreement to determine the level of consistency among volunteers’ rankings.

### In vitro protocol for white cast measurement

Artificial skin (VITRO-SKIN) was cut into 6.0 x 6.0 cm square pieces and then hydrated according to the manufacturer’s instructions (IMS, Florida, USA) 16–24 hours before the experiment. Each test formulation was applied to a distinct piece of VITRO-SKIN. The VITRO-SKIN was inserted into a slide mount and placed on top of a black acrylic sheet to produce a dark background before taking baseline color measurements. Three CIEL*a*b* measurements were taken per VITRO-SKIN at the top left, middle, and bottom right of the substrate (S6 Fig). Once the baseline measurements were taken, 2 mg/cm2 of the test samples were applied to their corresponding VITRO-SKIN. Post-application measurements were taken after a 15-minute rest to mimic usual sunscreen directions for human use. Once the formulations were set, three CIEL*a*b* measurements per sample were taken, similar to the baseline measurements. Moreover, the same procedure was performed with a white acrylic sheet as the background and reported separately. A flowchart summarizing the methodology can be found in S3 Appendix.

### Photography

Top-view photographs of the inner forearms and VITRO-SKIN were taken with an iPhone (X model) from a distance of 23 inches at 1x magnification, positioned directly above a studio light box with a blue background (Puluz Photo Light Box, Model PU5040). The photographs were captured through a top opening in the light box, under optimal lighting conditions provided by 144 built-in LED 25 lumen output 2835 lamp beads, with a Color Rendering Index (CRI) ≥ 90 and a color temperature of 6500K, before and after the application of the test samples, as illustrated in Fig 2A and 2B. The before and after photographs included a color correction card (ColorChecker Classic by Calibrite LLC, Wilmington, Delaware, USA), which was later used to white balance the images with the help of a photo-editing app (Snapseed by Google version 2.22, Mountain View, California, USA).

### Expert grading

Three expert graders were trained using a standardized white cast scale developed for this study (S2 Appendix). Training included assessments on practice forearm photographs (not study volunteers) and VITRO-SKIN. Following training, the expert graders independently evaluated and ranked the test formulations from least to most white cast. Graders assessed photographs of both the study volunteers’ forearms against a blue background and VITRO-SKIN samples against a black background, as described previously. Rankings were analyzed to determine inter-rater reliability. Intraclass correlation coefficients (ICC) and the corresponding 95% confidence intervals were calculated using SPSS Version 30.0.0 (IBM Corp., Armonk, NY, USA), employing a two-way mixed-effects model with absolute agreement to determine consistency among expert evaluations.

### White cast score

The white cast score for each test formulation was calculated according to the equation: $White\;Cast\;Score\;=\;\frac{L*f\;-\;L*i}{100\;-\;L*i}$ where L*f  =  L* measurement after test formulation application, and L*i  =  L* measurement before test formulation application. As part of the subjective questionnaire (S1 Appendix), participants responded to two key items: “What is the highest level of white cast you are willing to accept in exchange for sun protection (SPF)?” and “Please rank the different sunscreen formulations tested on your skin from least to most white cast using the line scale.” These responses were analyzed to determine in vivo preferences across all Fitzpatrick skin types, and intraclass correlation coefficients (ICC) were calculated to assess agreement among volunteers. By combining these data, we established and proposed the following interpretation thresholds for the white cast score: “No white cast”: ZnO 0%, ranked lowest in perceived whiteness across all skin types for face and body. “Reasonable”: ZnO 5%, consistently ranked as the most acceptable formulation for facial use after ZnO 0%. “Threshold”: ZnO 10% and benchmark (BM) formulations, which showed mixed acceptability depending on skin type. “Unacceptable”: ZnO 20–30%, ranked highest in perceived whiteness and least preferred across all skin types for face and body.

### Method validation and fit

The following set of analytical tests was conducted to validate our protocols. Linearity, accuracy, and precision for all CIEL*a*b* data were tested and calculated. The sample sizes refer to individual L* measurements for Fig 1 and Tables 1–3. For in vitro studies, we tested for both intra- and inter-day precision to confirm the repeatability and reliability of our measurements on different days with different staff. For in vivo studies, we tested only inter-day precision for the convenience of the study volunteers. We then conducted a comprehensive range of statistical tests, including the Shapiro-Wilk normality test, Pearson and Spearman Rho correlation, and Adjusted R-squared, to ensure the fit of our analysis. Validation tests followed the International Council for Harmonisation of Technical Requirements for Pharmaceuticals for Human Use (ICH) harmonized guideline Q2(R2) [27]. We used SPSS Version 30.0.0 for these statistics (IBM, Armonk, New York, USA). An 80–120% accuracy, a precision of < 10% RSD, and an adjusted R^2^ of > 0.650 were deemed acceptable for our studies.

### Other statistical analyses

The following set of descriptive statistics were provided for age: mean, SD, minimum, and maximum. Similarly, for gender, Fitzpatrick skin type, ethnicity and/or race, a detailed set of descriptive statistics is presented, along with their frequency expressed as number and/or percentage. Quantitative means and standard error of mean were calculated for the CIEL*a*b* data. In this study, we developed a white cast score to quantify the amount of white cast caused by sunscreens. For these white cast scores, the Zdifference (Zdiff) was calculated to determine whether correlation coefficients are equivalent or statistically different. First, we converted a pair of Pearson correlation coefficients into Fisher’s Z-scores to make them uniformly distributed. Then, we found the difference between these scores and adjusted for the variability in sample sizes to account for uncertainty. A larger Zdiff indicates a greater difference between the correlations when comparing this value to the critical threshold of Z = 1.96 (for 95% confidence). Graphs with mean, SEM, Kruskal-Wallis with Dunn’s post hoc tests and Wilcoxon Signed Rank Tests were done using PRISM 10 (GraphPad, Boston, Massachusetts, USA). We used R Studio Version 2024.09 (Posit PBC, Boston, Massachusetts, USA) and the RStudio package ggplot2, a powerful and widely used data visualization package in R, to generate the ITA° graph.

## Supporting information

S1 Fig Individual Typology Angle Subtypes of Volunteers. A total of thirteen (13) volunteers completed the study. The ITA°s range from Very Light to Brown subtypes.
(PDF)

S2 Fig In vitro Method of VITRO-SKIN over White Background is Less Sensitive to L* Value Changes Caused by Zinc Oxide Formulations. (A) ZnO 0% and 5% test formulations show statistically significant changes in L* values in comparison with 20% and 30% ZnO formulations after their application (** = p < 0.001 and *** = p < 0.001) n = 6. (B) Adjusted R^2^ was calculated to indicate the goodness of fit. VITRO-SKIN on white acrylic’s L* measurements (n = 30) ranged from 86 to 90. (C) Same graph as (b) but zoomed-in to observe L* value measurements clearly. BM = benchmark; ns = not significant. Error bars are shown as mean ± SEM.
(PDF)

S3 Fig Visual Comparison of Test Formulations Applied on VITRO-SKIN over Black Background. Representative images of VITRO-SKIN before test formulation application and after test formulations containing increasing concentrations of ZnO (0%, 5%, 10%, 20%, 30%, and benchmark) applied to VITRO-SKIN placed over a black acrylic background. A progressive increase in visible whiteness is clearly observed with higher ZnO percentages, highlighting the enhanced contrast provided by the dark background.
(PDF)

S4 Fig Visual Comparison of Test Formulations Applied on VITRO-SKIN over White Background. Representative images of VITRO-SKIN before test formulation application and after test formulations containing increasing concentrations of ZnO (0%, 5%, 10%, 20%, 30%, and benchmark) applied to VITRO-SKIN placed over a white acrylic background. In contrast to the black background setup, differences in visible whiteness among ZnO percentages are minimal and difficult to distinguish, suggesting that a white background may obscure subtle variations in white cast.
(PDF)

S5 Fig L* Value Increases after Application of Zinc Oxide Formulations. In vivo: (A) Test formulations show statistically significant changes in L* values after their use in All Volunteers (**** p < 0.0001, n = 39). (B-D) Light skin (n = 15), medium skin (n = 9) and dark skin pigmentation (n = 15) volunteers show statistically significant changes in L* value after applying ZnO test formulations (** p < 0.01 and **** p < 0.0001). (E-F) In vitro: Test formulations have statistically significant difference after their application (* p < 0.05). n = 6. Error bars are shown as mean ± SEM.
(PDF)

S6 Fig Materials and Methods for Taking CIEL*a*b* Measurements. (A) Areas where CIEL*a*b* measurements were taken on VITRO-SKIN for the in vitro protocol and inside the silicone squares for the in vivo protocol. (B) Image of the NIX Pro 2 Color Sensor and App from the vendor’s website: https://www.nixsensor.com/product/nix-pro-color-sensor/.
(PDF)

S1 Table Inter-day Precision of In Vivo L* Measurements by Zinc Oxide Percentage. (PDF)

S2 Table Inter- and Intra-Day Precision of In Vitro L* Measurements by Zinc Oxide Percentage. (PDF)

S3 Table Demographics of the 13 Volunteers who Completed the In Vivo Protocol. (PDF)

S4 Table Laboratory Zinc Oxide Test Sample Formulations. (PDF)

S5 Table Commercially available Broad Spectrum SPF 30 with 14.7% ZnO. (PDF)

S1 Appendix Subjective Questionnaire for Thirteen Volunteers. (PDF)

S2 Appendix White cast Training Color Scale. (PDF)

S3 Appendix Methods Flowchart. (PDF)
