# Supplementary figures and images for: A standardized scoring method for measuring white cast of mineral sunscreens and improving user compliance across diverse skin tones
Source: PLoS One. 2025 Aug 26;20(8):e0319891. doi: 10.1371/journal.pone.0319891 (PMC12380271; doi:10.1371/journal.pone.0319891)

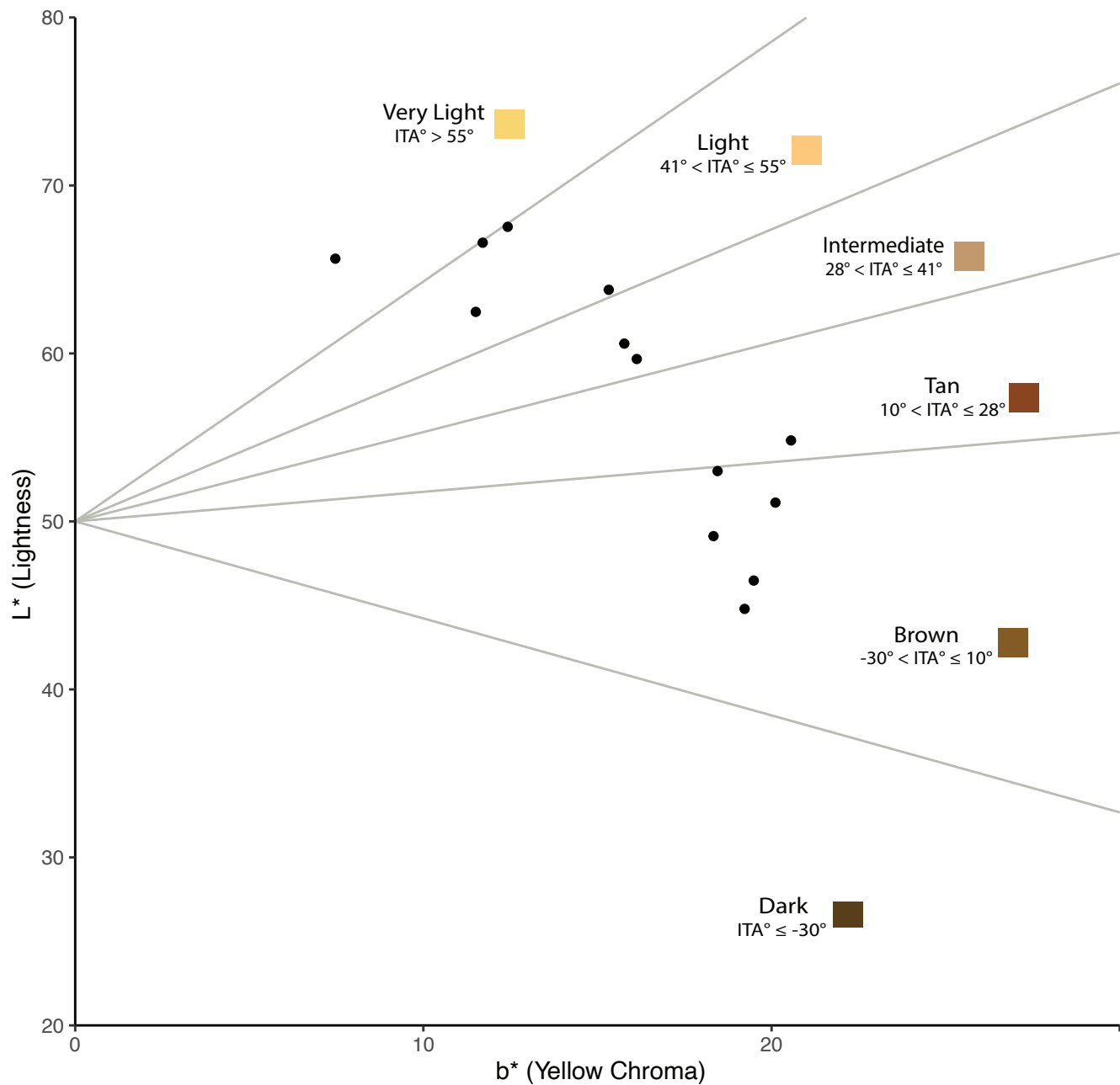

Supplement: S1 Fig — A total of thirteen (13) volunteers completed the study. The ITA°s range from Very Light to Brown subtypes. (PDF) [file pone.0319891.s001.pdf]

(A)

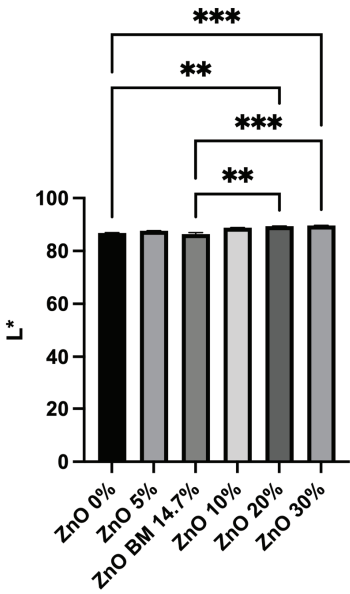

Kruskal-Wallis p value < 0.0001

(B)

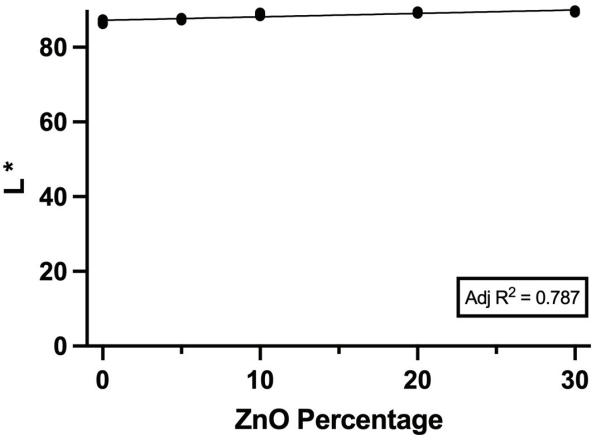

(C)

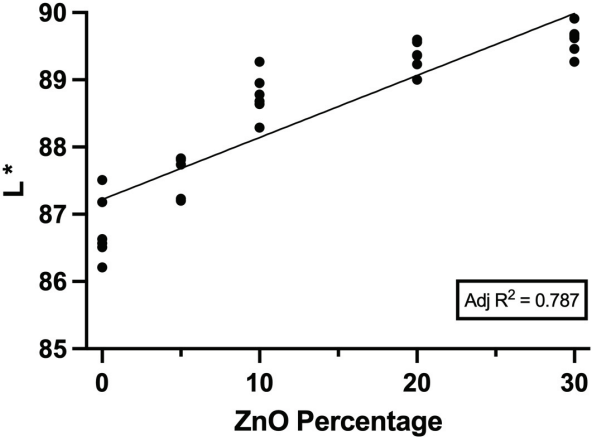

Supplement: S2 Fig — (A) ZnO 0% and 5% test formulations show statistically significant changes in L* values in comparison with 20% and 30% ZnO formulations after their application (** = p < 0.001 and *** = p < 0.001) n = 6. (B) Adjusted R2 was calculated to indicate the goodness of fit. VITRO-SKIN on white acrylic’s L* measurements (n = 30) ranged from 86 to 90. (C) Same graph as (b) but zoomed-in to observe L* value measurements clearly. BM = benchmark; ns = not significant. Error bars are shown as mean ± SEM. (PDF) [file pone.0319891.s002.pdf]

**Before Application**

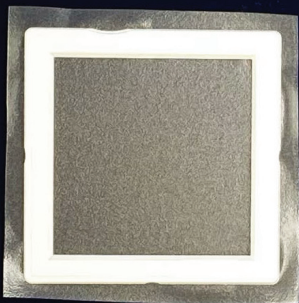

**BM ZnO 14.7%**

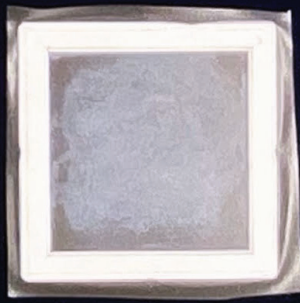

**ZnO 0%**

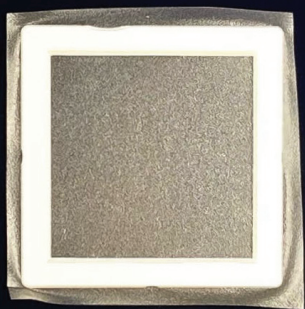

**ZnO 5%**

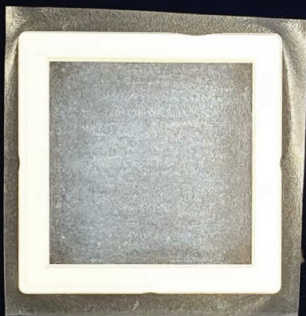

**ZnO 10%**

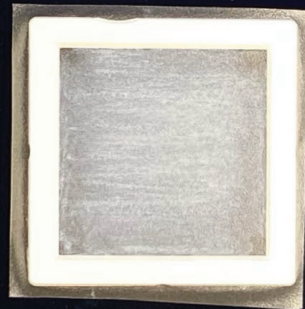

**ZnO 20%**

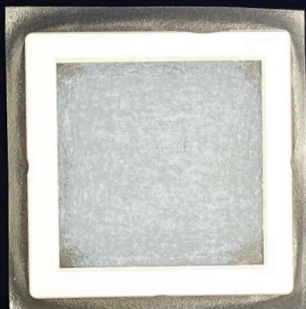

**ZnO 30%**

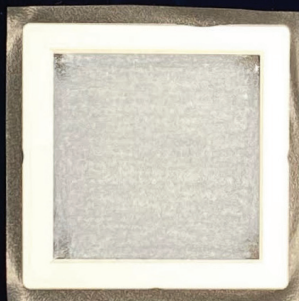

Supplement: S3 Fig — Representative images of VITRO-SKIN before test formulation application and after test formulations containing increasing concentrations of ZnO (0%, 5%, 10%, 20%, 30%, and benchmark) applied to VITRO-SKIN placed over a black acrylic background. A progressive increase in visible whiteness is clearly observed with higher ZnO percentages, highlighting the enhanced contrast provided by the dark background. (PDF) [file pone.0319891.s003.pdf]

**Before Application**

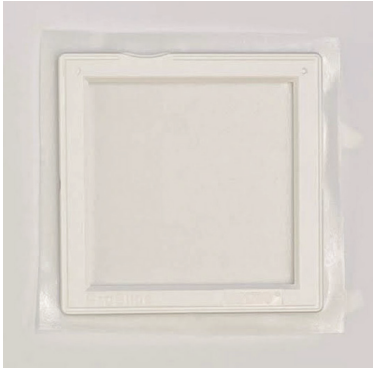

**BM ZnO 14.7%**

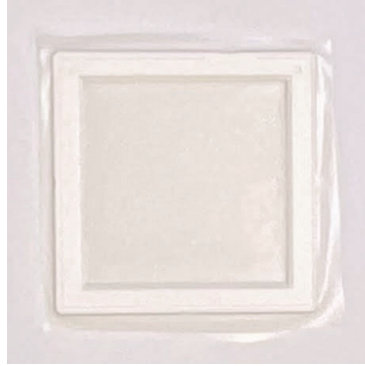

**ZnO 0%**

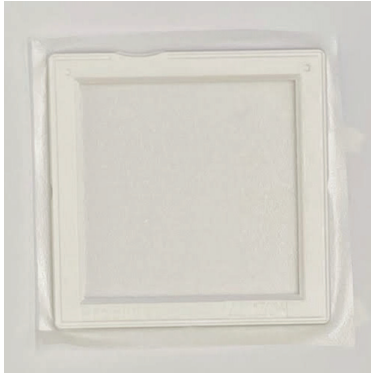

**ZnO 5%**

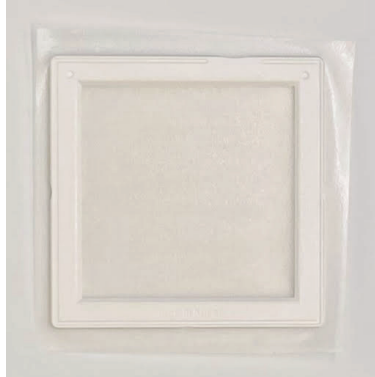

**ZnO 10%**

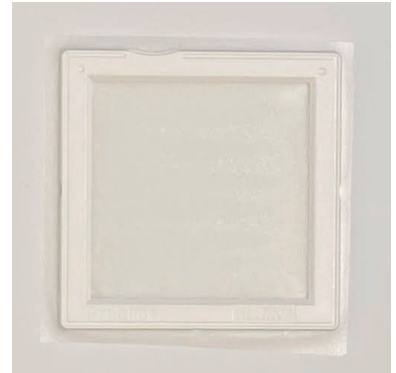

**ZnO 20%**

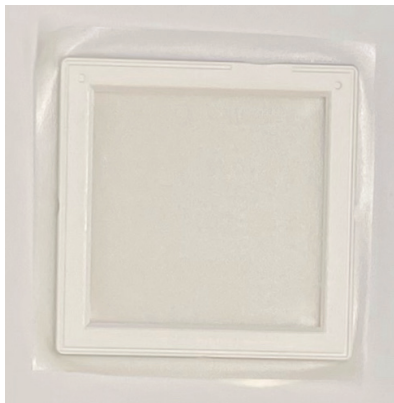

**ZnO 30%**

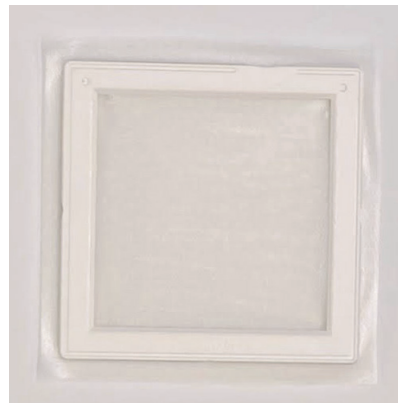

Supplement: S4 Fig — Representative images of VITRO-SKIN before test formulation application and after test formulations containing increasing concentrations of ZnO (0%, 5%, 10%, 20%, 30%, and benchmark) applied to VITRO-SKIN placed over a white acrylic background. In contrast to the black background setup, differences in visible whiteness among ZnO percentages are minimal and difficult to distinguish, suggesting that a white background may obscure subtle variations in white cast. (PDF) [file pone.0319891.s004.pdf]

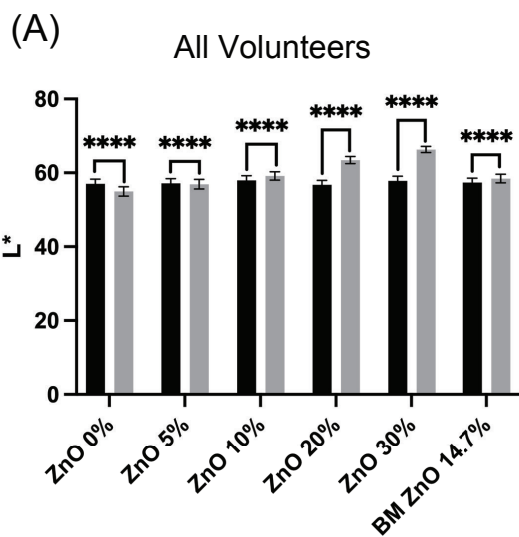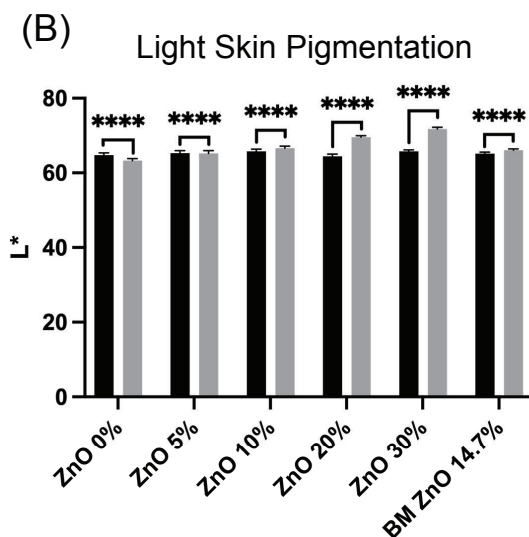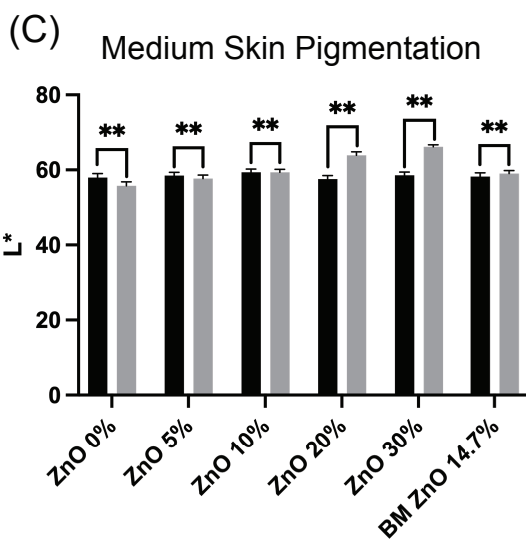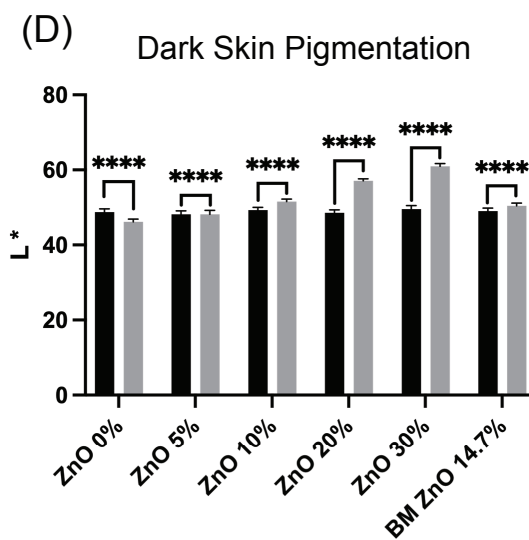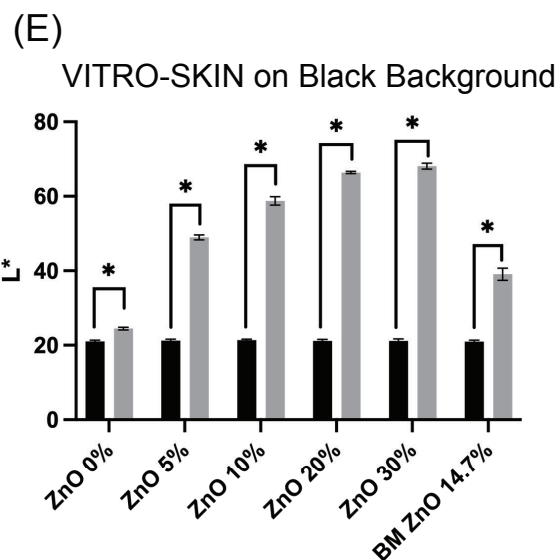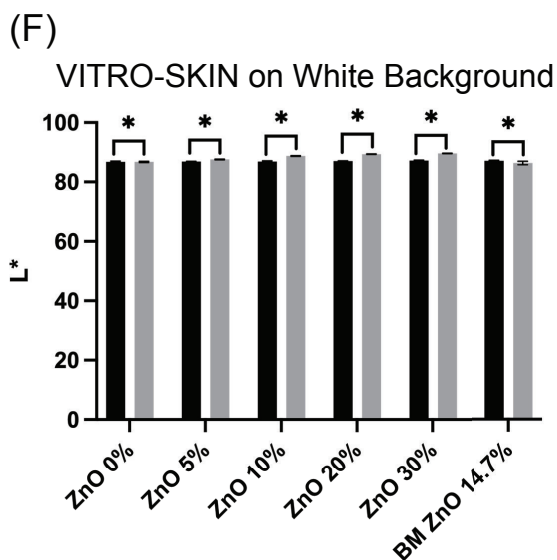

■ Before Application  
■ After Application

Supplement: S5 Fig — In vivo: (A) Test formulations show statistically significant changes in L* values after their use in All Volunteers (**** p < 0.0001, n = 39). (B-D) Light skin (n = 15), medium skin (n = 9) and dark skin pigmentation (n = 15) volunteers show statistically significant changes in L* value after applying ZnO test formulations (** p < 0.01 and **** p < 0.0001). (E-F) In vitro: Test formulations have statistically significant difference after their application (* p < 0.05). n = 6. Error bars are shown as mean ± SEM. (PDF) [file pone.0319891.s005.pdf]

(A)

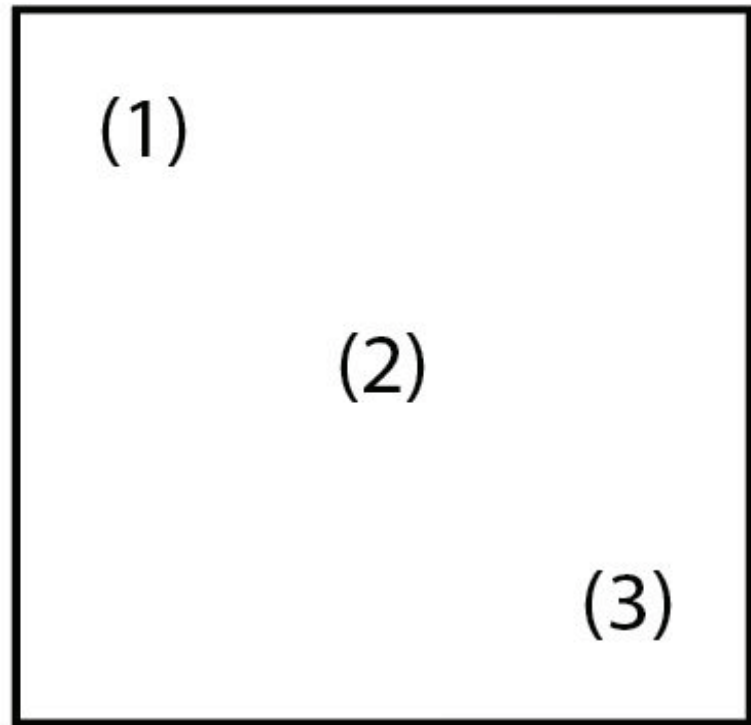

(B)

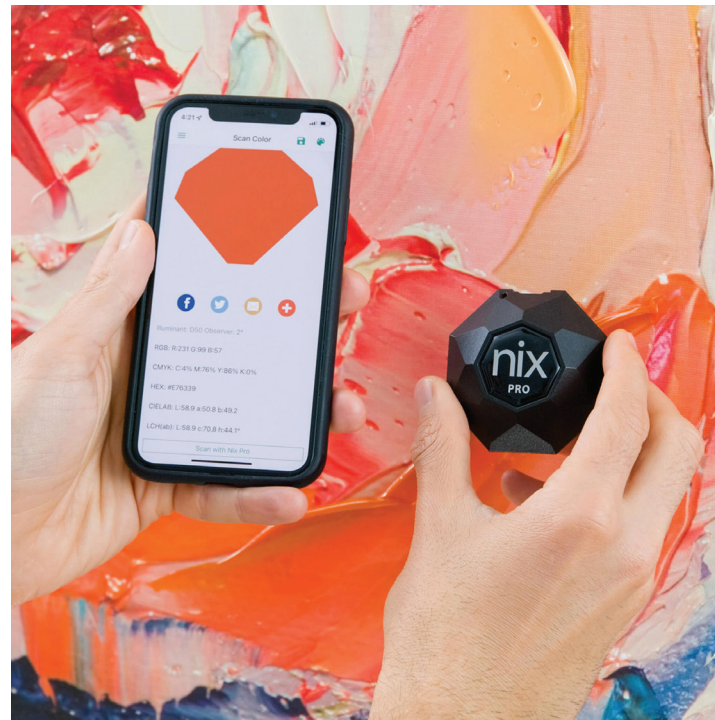

Supplement: S6 Fig — (A) Areas where CIEL*a*b* measurements were taken on VITRO-SKIN for the in vitro protocol and inside the silicone squares for the in vivo protocol. (B) Image of the NIX Pro 2 Color Sensor and App from the vendor’s website: https://www.nixsensor.com/product/nix-pro-color-sensor/. (PDF) [file pone.0319891.s006.pdf]

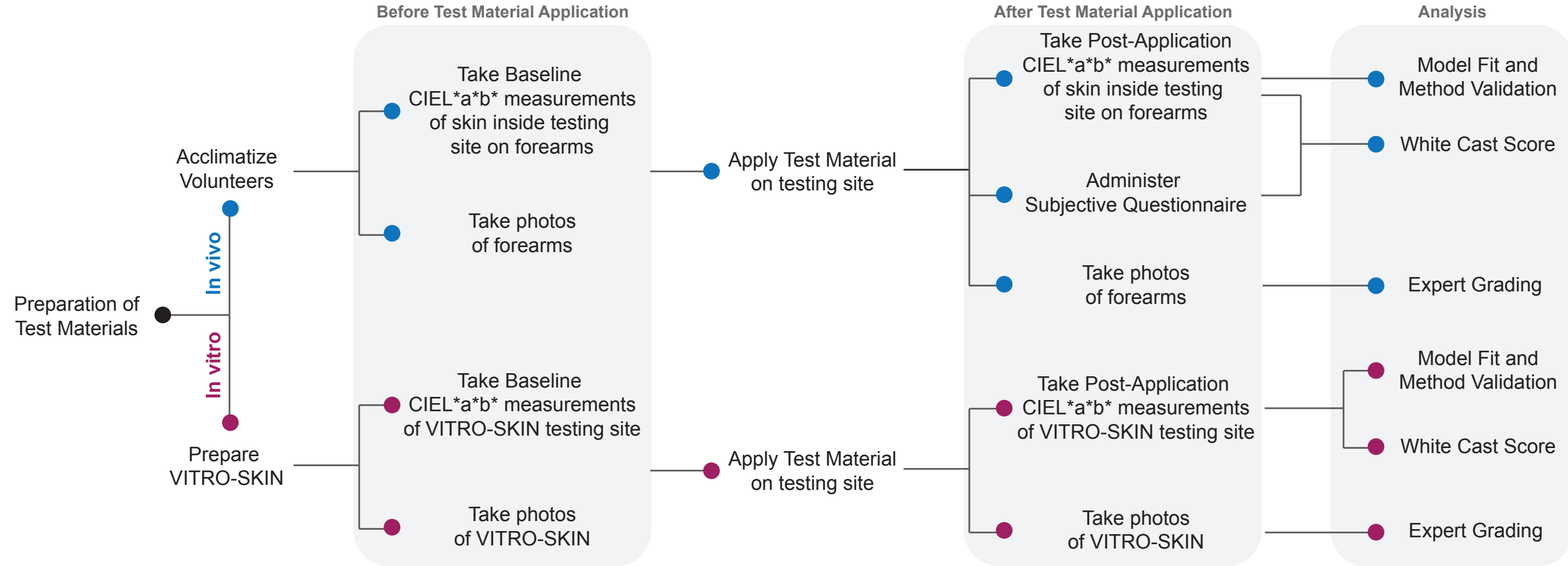

Supplement: S3 Appendix — (PDF) [file pone.0319891.s014.pdf]
