# Supplementary material for: A standardized scoring method for measuring white cast of mineral sunscreens and improving user compliance across diverse skin tones
Source: PLoS One. 2025 Aug 26;20(8):e0319891. doi: 10.1371/journal.pone.0319891 (PMC12380271; doi:10.1371/journal.pone.0319891)
Supplement: S1 Table — (PDF) [file pone.0319891.s007.pdf]

**S1 Table. Inter-day Precision of In Vivo L* Measurements by Zinc Oxide Percentage**

| Factor | ZnO % | % RSD Before Sample Application | % RSD After Sample Application |
|---|---|---|---|
| Very Light Subtype ITA° | 0 | 0.8 | 0.99 |
| | 5 | 1.33 | 1.33 |
| | 10 | 1.48 | 0.4 |
| | 20 | 0.96 | 0.66 |
| | 30 | 0.57 | 1.56 |
| Light Subtype ITA° | 0 | 3.92 | 3.56 |
| | 5 | 4.31 | 5.02 |
| | 10 | 3.79 | 3.52 |
| | 20 | 3.99 | 2.98 |
| | 30 | 2.41 | 2.23 |
| Intermediate Subtype ITA° | 0 | 2.21 | 1.37 |
| | 5 | 2.08 | 1.62 |
| | 10 | 2.81 | 2.96 |
| | 20 | 1.71 | 4.77 |
| | 30 | 1.05 | 5.31 |
| Tan Subtype ITA° | 0 | 0.73 | 1.11 |
| | 5 | 0.97 | 0.8 |
| | 10 | 0.85 | 2.45 |
| | 20 | 2.8 | 0.76 |
| | 30 | 1.1 | 1.32 |
| Brown Subtype ITA° | 0 | 6.68 | 6.27 |
| | 5 | 6.85 | 8.55 |
| | 10 | 6.46 | 5.11 |
| | 20 | 8.04 | 3.51 |
| | 30 | 9.28 | 4.75 |
| Light Skin Pigmentation | 0 | 3.54 | 3.36 |
| | 5 | 3.87 | 4.52 |
| | 10 | 3.42 | 3.23 |
| | 20 | 3.64 | 2.07 |
| | 30 | 2.2 | 2.44 |
| Medium Skin Pigmentation | 0 | 5.54 | 5.92 |
| | 5 | 4.71 | 5.11 |
| | 10 | 4.18 | 3.35 |
| | 20 | 5.66 | 4.52 |
| | 30 | 4.69 | 4.25 |
| Dark Skin Pigmentation | 0 | 6.68 | 6.27 |
| | 5 | 6.85 | 8.55 |
| | 10 | 6.46 | 5.11 |
| | 20 | 8.04 | 3.51 |
| | 30 | 9.28 | 4.75 |

All RSDs were reportedly < 10%.
