# Supplementary material for: A standardized scoring method for measuring white cast of mineral sunscreens and improving user compliance across diverse skin tones
Source: PLoS One. 2025 Aug 26;20(8):e0319891. doi: 10.1371/journal.pone.0319891 (PMC12380271; doi:10.1371/journal.pone.0319891)
Supplement: S2 Table — (PDF) [file pone.0319891.s008.pdf]

S2 Table. Inter- and Intra-Day Precision of In Vitro L* Measurements by Zinc Oxide Percentage

Factor		ZnO %	% RSD Before Sample Application	% RSD After Sample Application
Inter-Day	Black Acrylic	0	3.79	3.57
		5	4.77	3.41
		10	3.20	4.76
		20	4.57	1.11
		30	6.41	2.93
	White Acrylic	0	0.73	0.55
		5	0.39	0.35
		10	0.58	0.36
		20	0.39	0.25
		30	0.50	0.24
Intra-Day 1	Black Acrylic	0	1.29	3.15
		5	3.86	1.75
		10	2.21	1.63
		20	2.66	1.22
		30	0.71	1.26
	White Acrylic	0	0.13	0.07
		5	0.29	0.05
		10	0.16	0.24
		20	0.13	0.34
		30	0.21	0.13
Intra-Day 2	Black Acrylic	0	1.65	1.32
		5	1.78	5.10
		10	1.25	6.85
		20	2.17	0.12
		30	4.30	0.26
	White Acrylic	0	1.15	0.78
		5	0.29	0.41
		10	0.70	0.28
		20	0.08	0.19
		30	0.43	0.36

All RSDs were reportedly < 10%.
