# Supplementary material for: A standardized scoring method for measuring white cast of mineral sunscreens and improving user compliance across diverse skin tones
Source: PLoS One. 2025 Aug 26;20(8):e0319891. doi: 10.1371/journal.pone.0319891 (PMC12380271; doi:10.1371/journal.pone.0319891)
Supplement: S3 Table — (PDF) [file pone.0319891.s009.pdf]

S3 Table. Demographics of the 13 Volunteers who Completed the In Vivo Protocol

Parameter		n	%
Sex	Female	13	100
	Male	0	0
Race & Ethnicity	Asian	2	15.38
	Black	5	38.46
	Caucasian	4	30.77
	Hispanic or Latino	2	15.38
Fitzpatrick Skin Type	I	0	0
	II	4	30.77
	III	3	23.08
	IV	1	7.69
	V	3	23.08
	VI	2	15.38
Individual Typology Angle Subtype	Very Light	1	7.69
	Light	4	30.77
	Intermediate	2	15.38
	Tan	1	7.69
	Brown	5	38.46
	Dark	0	0
Age	Mean ± SD	35 ± 15	
	Min	20	
	Max	60	
