# Supplementary material for: A standardized scoring method for measuring white cast of mineral sunscreens and improving user compliance across diverse skin tones
Source: PLoS One. 2025 Aug 26;20(8):e0319891. doi: 10.1371/journal.pone.0319891 (PMC12380271; doi:10.1371/journal.pone.0319891)
Supplement: S4 Table — (PDF) [file pone.0319891.s010.pdf]

S4 Table. Laboratory Zinc Oxide Test Sample Formulations

Ingredients (INCI Name)	wt. %				
	0% ZnO	5% ZnO	10% ZnO	20% ZnO	30% ZnO
Zinc Oxide (and) Triethoxycaprylylsilane	0	5.05	10.1	20.2	30.3
Kaolin	5.05	0	0	0	0
Ricinus Communis (Castor) Seed Oil	43.45	43.45	38.4	28.3	18.2
Helianthus Annuus (Sunflower) Seed Oil	10	10	10	10	10
Sorbitan Laurate (and) Polyglyceryl-4 Laurate (and) Dilauryl Citrate	0.5	0.5	0.5	0.5	0.5
Butyrospermum Parkii (Shea) Butter	40	40	40	40	40
Stearic acid	1	1	1	1	1
