# Supplementary material for: A standardized scoring method for measuring white cast of mineral sunscreens and improving user compliance across diverse skin tones
Source: PLoS One. 2025 Aug 26;20(8):e0319891. doi: 10.1371/journal.pone.0319891 (PMC12380271; doi:10.1371/journal.pone.0319891)
Supplement: S5 Table — (PDF) [file pone.0319891.s011.pdf]

S5 Table. Commercially available Broad Spectrum SPF 30 with 14.7% ZnO

Ingredients (INCI Name)
Zinc Oxide (14.7%)
Water
Cocos Nucifera (Coconut) Oil
Butyloctyl Salicylate
Diheptyl Succinate
Capryloyl Glycerin/Sebacic Acid Copolymer
Methyl Dihydroabietate
Butyrospermum Parkii (Shea) Butter
Cetearyl Alcohol
Sodium Stearoyl Glutamate
Theobroma Cacao (Cocoa) Seed Butter
Tocopherol
Coco-glucoside
Microcrystalline Cellulose
Cetyl Alcohol
Behenyl Alcohol
Bisabolol
Arachidyl Glucoside
Phenozyethanol
Arachidyl Alcohol
Cellulose Gum
Sodium Hyaluronate
Sodium Gluconate
Citric Acid
Ethylhexylglycerin
Polyhydroxystearic Acid
