# Supplementary material for: A standardized scoring method for measuring white cast of mineral sunscreens and improving user compliance across diverse skin tones
Source: PLoS One. 2025 Aug 26;20(8):e0319891. doi: 10.1371/journal.pone.0319891 (PMC12380271; doi:10.1371/journal.pone.0319891)
Supplement: S1 Appendix — (PDF) [file pone.0319891.s012.pdf]

GOOD MOLECULES

SEE A DIFFERENCE IN YOUR SKIN

Your invaluable insights on mineral sunscreen white cast and its impact on your skin are crucial for our research.

Volunteer ID: ________________________

1. How would you describe the appearance of white cast on your skin when using mineral sunscreen? Please share your thoughts in detail. (Open question)

____

____

____

____

2. I am concerned about white cast. (Choose one)

_______ Strongly Agree _______ Agree _______ Disagree _______ Strongly Disagree

3. What is the highest level of white cast you are willing to trade for sun protection (SPF)? (Choose one for Body[B] and one for Face [F])

_______ Sunscreen Formulation 1 _______ Sunscreen Formulation 2
_______ Sunscreen Formulation 3 _______ Sunscreen Formulation 4
_______ Sunscreen Formulation 5 _______ Sunscreen Formulation 6

4. Please rank the different sunscreen formulations tested on your skin from least to most white cast using the line scale:

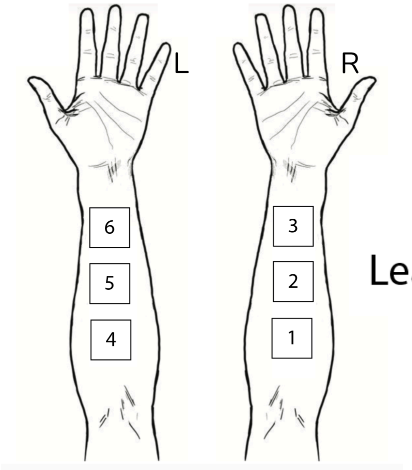

Least Whitecast — Most Whitecast
