# Supplementary material for: A standardized scoring method for measuring white cast of mineral sunscreens and improving user compliance across diverse skin tones
Source: PLoS One. 2025 Aug 26;20(8):e0319891. doi: 10.1371/journal.pone.0319891 (PMC12380271; doi:10.1371/journal.pone.0319891)
Supplement: S2 Appendix — (PDF) [file pone.0319891.s013.pdf]

		Black Scale						Yellow Scale		
1	2	3	4	5	6	5	4	3	2	1
Pure Black					Pure White					Pure Yellow
**NO WHITE CAST**										**NO WHITE CAST**
**Color**	**Color**	**Color**	**Color**	**Color**	**Color**	**Color**	**Color**	**Color**	**Color**	**Color**
sRGB (0 to 255): 0 0 0	sRGB (0 to 255): 235 235 235	sRGB (0 to 255): 241 241 241	sRGB (0 to 255): 246 246 246	sRGB (0 to 255): 252 252 252	sRGB (0 to 255): 255 255 255	sRGB (0 to 255): 255 255 248	sRGB (0 to 255): 254 253 246	sRGB (0 to 255): 253 252 242	sRGB (0 to 255): 252 250 237	sRGB (0 to 255): 255 255 0
**HEX (#): 000000**	**HEX (#): EBEBEB**	**HEX (#): F1F1F1**	**HEX (#): F6F6F6**	**HEX (#): FCFCFC**	**HEX (#): FFFFFF**	**HEX (#): FFFFF8**	**HEX (#): FEFDF6**	**HEX (#): FDFCF2**	**HEX (#): FCFAED**	**HEX (#): FFFF00**
CMYK (0% to 100%): 75% 68% 67% 90%	CMYK (0% to 100%): 7% 5% 5% 0%	CMYK (0% to 100%): 4% 3% 3% 0%	CMYK (0% to 100%): 2% 1% 1% 0%	CMYK (0% to 100%): 0% 0% 0% 0%	CMYK (0% to 100%): 0% 0% 0% 0%	CMYK (0% to 100%): 0% 0% 2% 0%	CMYK (0% to 100%): 0% 0% 3% 0%	CMYK (0% to 100%): 0% 0% 4% 0%	CMYK (0% to 100%): 1% 1% 7% 0%	CMYK (0% to 100%): 6% 0% 97% 0%
CIELAB (0 to 100, -128 to 128, -128 to 128): 0.00 0.00 0.00	CIELAB (0 to 100, -128 to 128, -128 to 128): 93.00 0.00 0.00	CIELAB (0 to 100, -128 to 128, -128 to 128): 95.00 0.00 0.00	CIELAB (0 to 100, -128 to 128, -128 to 128): 97.00 0.00 0.00	CIELAB (0 to 100, -128 to 128, -128 to 128): 99.00 0.00 0.00	CIELAB (0 to 100, -128 to 128, -128 to 128): 100.00 0.00 0.00	CIELAB (0 to 100, -128 to 128, -128 to 128): 99.86 -0.90 3.35	CIELAB (0 to 100, -128 to 128, -128 to 128): 99.24 -0.56 3.48	CIELAB (0 to 100, -128 to 128, -128 to 128): 98.84 -0.93 4.93	CIELAB (0 to 100, -128 to 128, -128 to 128): 98.16 -0.96 6.50	CIELAB (0 to 100, -128 to 128, -128 to 128): 97.61 -15.75 93.39

GOOD MOLECULES

SEE A DIFFERENCE IN YOUR SKIN
